# Tetanus disease and deaths in men reveal need for vaccination

**DOI:** 10.2471/BLT.15.166777

**Published:** 2016-06-02

**Authors:** Shona Dalal, Julia Samuelson, Jason Reed, Ahmadu Yakubu, Buhle Ncube, Rachel Baggaley

**Affiliations:** a Department of HIV/AIDS, World Health Organization, 20 Avenue Appia, 1211 Geneva, Switzerland.; b Office of the Global AIDS Coordinator, United States Department of State, Washington DC, United States of America.; c Department of Immunization, Vaccines and Biologicals, World Health Organization, Geneva, Switzerland.; d Department of HIV, TB and Hepatitis, Communicable Diseases Cluster, World Health Organization Regional Office, Harare, Zimbabwe.

## Abstract

With efforts focused on the elimination of maternal and neonatal tetanus, less attention has been given to tetanus incidence and mortality among men. Since 2007 voluntary medical male circumcision has been scaled-up in 14 sub-Saharan African countries as an effective intervention to reduce the risk of human immunodeficiency virus (HIV) acquisition among men. As part of a review of adverse events from these programmes, we identified 13 cases of tetanus from five countries reported to the World Health Organization (WHO) up to March 2016. Eight patients died and only one patient had a known history of tetanus vaccination. Tetanus after voluntary medical male circumcision was rare among more than 11 million procedures conducted. Nevertheless, the cases prompted a review of the evidence on tetanus vaccination coverage and case notifications in sub-Saharan Africa, supplemented by a literature review of non-neonatal tetanus in Africa over the years 2003–2014. The WHO African Region reported the highest number of non-neonatal tetanus cases per million population and lowest historic coverage of tetanus-toxoid-containing vaccine. Coverage of the third dose of diphtheria–tetanus–polio vaccine ranged from 65% to 98% across the 14 countries in 2013. In hospital-based studies, non-neonatal tetanus comprised 0.3–10.7% of admissions, and a median of 71% of patients were men. The identification of tetanus cases following voluntary medical male circumcision highlights a gender gap in tetanus morbidity disproportionately affecting men. Incorporating tetanus vaccination for boys and men into national programmes should be a priority to align with the goal of universal health coverage.

## Résumé

Avec l'orientation des efforts sur l'élimination du tétanos maternel et néonatal, une moindre attention a été portée sur l'incidence et la mortalité du tétanos dans la population masculine. Depuis 2007, la circoncision médicale masculine volontaire s'est intensifiée dans 14 pays d'Afrique subsaharienne, en tant qu'intervention efficace pour réduire le risque d'acquisition du virus de l'immunodéficience humaine (VIH) chez les hommes. Dans le cadre d'une analyse des effets indésirables de ces programmes, nous avons identifié 13 cas de tétanos, dans cinq pays, qui ont été notifiés à l'Organisation mondiale de la Santé (OMS) jusqu'à mars 2016. Huit patients sont décédés et un seul patient avait un antécédent connu de vaccination antitétanique. Sur plus de 11 millions de procédures réalisées, les infections tétaniques suite à une circoncision médicale masculine volontaire ont été rares. Néanmoins, ces cas d'infection nous ont poussés à mener une étude des données disponibles sur la couverture antitétanique et sur la notification des cas en Afrique subsaharienne, complétée par une revue de la littérature sur le tétanos non-néonatal en Afrique sur la période de 2003 à 2014. Sur la période étudiée, les pays répertoriés dans la Région africaine de l'OMS correspondent au plus grand nombre de cas de tétanos non-néonatal pour un million d'habitants et à la plus faible couverture vaccinale par anatoxine tétanique. En 2013, dans les 14 pays considérés, le taux d'administration de la troisième dose du vaccin diphtérie-tétanos-poliomyélite se situait entre 65% et 98%. Selon les études réalisées dans des hôpitaux, le tétanos non néonatal est responsable de 0,3 à 10,7% des admissions, pour lesquelles 71% des patients, en moyenne, sont des hommes. L'identification des cas de tétanos déclarés après une circoncision médicale masculine volontaire a permis de révéler une disparité homme-femme en termes de mortalité, en défaveur des hommes. L'intégration de la vaccination antitétanique des garçons et des hommes dans les programmes nationaux devrait être une priorité pour poursuivre l'objectif de couverture sanitaire universelle.

## Resumen

Dado que se han concentrado los esfuerzos en la eliminación del tétanos materno y neonatal, se ha prestado menos atención a la incidencia y mortalidad del tétanos en los hombres. Desde 2007, ha aumentado la circuncisión médica masculina voluntaria en 14 países subsaharianos, puesto que se trata de una intervención efectiva para reducir el riesgo de contagio del virus de la inmunodeficiencia humana (VIH) en los hombres. Como parte de una revisión de los fenómenos adversos derivados de estos programas, se identificaron 13 casos de tétanos de cinco países notificados a la Organización Mundial de la Salud (OMS) hasta marzo de 2016. Ocho pacientes murieron y solo uno estaba vacunado contra el tétanos. Tras practicar la circuncisión médica masculina voluntaria, el tétanos era poco frecuente entre más de 11 millones de intervenciones realizadas. No obstante, los casos dieron lugar a una revisión de la prueba de la cobertura de vacunas contra el tétanos y las notificaciones de los casos en el África subsahariana, junto con una revisión documental del tétanos no neonatal en África durante los años 2003 a 2014. La OMS de la región africana informó del mayor número de casos de tétanos no neonatal por cada millón de habitantes y de la menor cobertura de la vacuna con toxoide tetánico de la historia. En 2013, la cobertura de la tercera dosis de la vacuna de la difteria, tétanos, polio abarcó de un 65% a un 98% en los 14 países. En estudios centrados en los hospitales, el tétanos no neonatal abarcó entre un 0,3% y un 10,7% de admisiones, y una media de 71% de los pacientes eran hombres. La identificación de los casos de tétanos tras la circuncisión médica masculina voluntaria destaca una diferencia desproporcionada entre hombres y mujeres en cuanto a la morbilidad por tétanos, en detrimento de los hombres. La incorporación de vacunas contra el tétanos para niños y hombres en programas nacionales debería ser prioritaria para ajustarse al objetivo de cobertura sanitaria universal.

## ملخص

أدى تركز الجهود على القضاء على مرض التيتانوس لدى الأمهات والأطفال حديثي الولادة إلى انحسار الاهتمام بحالات الإصابة بمرض التيتانوس وما يسببه من حالات الوفاة لدى الرجال. فقد ارتفع معدل ختان الذكور طوعيًا لأغراض طبية منذ عام 2007 في 14 دولة من الدول الأفريقية الواقعة جنوبي الصحراء الكبرى باعتباره تدخلًا فعالًا للحد من خطر انتقال مرض عوز المناعة البشري (HIV) بين الرجال. وقد حددنا 13 حالة للإصابة بمرض التيتانوس في خمس دول في إطار مراجعة الآثار السلبية الناتجة عن تلك البرامج، وذلك وفقًا لما ورد لمنظمة الصحة العالمية (WHO) حتى شهر مارس 2016، فقد توفى ثمانية من المرضى وكان لدى مريض واحد فقط تاريخًا معلومًا لتلقي لقاح التيتانوس. وكانت الإصابة بالتيتانوس عقب إجراء ختان الذكور طوعيًا لأغراض طبية نادرة الحدوث بين ما يزيد عن 11 مليون إجراء تم اتخاذه. وبالرغم من ذلك، تطلبت الحالات إجراء مراجعة للوقوف على الدلائل التي تشير إلى وجود تغطية للتطعيم ضد التيتانوس والإبلاغ عن حالات الإصابة بهذا المرض في الدول الأفريقية الواقعة جنوبي الصحراء الكبرى، مع دعم ذلك بمراجعة للمؤلفات الطبية التي تناولت حالات الإصابة بالتيتانوس في غير الأطفال حديثي الولادة بالدول الأفريقية على مدى الأعوام من 2003 حتى 2014. وقد وردت معلومات بأن منطقة الدول الأفريقية التي يشملها نطاق عمل منظمة الصحة العالمية سجلت أعلى عدد من حالات الإصابة بمرض التيتانوس في غير الأطفال حديثي الولادة لكل مجموعة سكانية يبلغ عدد أفرادها مليون نسمة وأقل معدل للتغطية في الفترة السابقة فيما يتعلق باللقاح المحتوي على ذوفان التيتانوس. وتراوحت نسبة تغطية الجرعة الثالثة من لقاح الدفتريا والتيتانوس وشلل الأطفال من 65% إلى 98% في 14 دولة في عام 2013. ووفقًا للدراسات القائمة على الحالات الموجودة بالمستشفيات، تألفت حالات الإصابة بالتيتانوس في غير الأطفال حديثي الولادة من 0.3% إلى 10.7% من حالات العلاج بالمستشفيات، وكان المرضى بمتوسط نسبة يبلغ 71% من الرجال. إن تحديد حالات الإصابة بمرض التيتانوس عقب إجراء ختان الذكور طوعيًا لأغراض طبية قد ألقى الضوء على وجود فجوة بين الجنسين فيما يتعلق بالإصابة بمرض التيتانوس بما يعكس تفاوتًا كبيرًا في معاناة الرجال من المرض. ويجب أن يمثل تضمين لقاح التيتانوس المقدم للبنين والرجال في البرامج الوطنية أحد الأولويات تماشيًا مع الهدف المتمثل في توفير التغطية الصحية على المستوى العالمي.

## 摘要

相对于对消除产妇和新生儿破伤风的高度重视，社会各界对男性破伤风发病和死亡的关注度较少。 自 2007 年开始，在 14 个撒哈拉以南的非洲国家中，男性自愿包皮环切手术作为一种可降低男性间艾滋病病毒 (HIV) 感染率的有效干预措施，已经被广为采用。 作为对此类计划不良事件审查的一部分，截至 2016 年 3 月，我们从五个向世界卫生组织 (WHO) 报告的国家中确定了 13 例破伤风患者。其中八名患者已经死亡，据了解，仅有一名患者接种过破伤风疫苗。 在 1,100 万例男性自愿包皮环切手术中，术后出现破伤风的情况很少。 然而，我们对这些病例启动了针对撒哈拉以南非洲国家破伤风疫苗接种覆盖面和病例通知的证据审查，并补充以针对 2003-2014 年非洲地区非新生儿破伤风的文献审查。世界卫生组织 (WHO) 非洲地区报告了每 100 万人口中的非新生儿破伤风病例的最高数量和含有破伤风类病毒的疫苗接种历史最低覆盖率。 2013 年，在 14 个国家中，第三剂量的白喉、破伤风、脊髓灰质炎疫苗覆盖率介于 65% 至 98% 之间。基于医院的研究显示，非新生儿破伤风中 0.3-10.7% 住院就诊，平均 71% 的患者为男性。 男性自愿包皮环切手术后确诊的破伤风病例突出显示了性别差距，即在男性中有着不成比例的高破伤风发病率。 应该将男孩和男性破伤风疫苗接种纳入国家计划作为优先事项，从而实现全民健康覆盖的目标。

## Случаи заболевания и смерти мужчин от столбняка показывают необходимость вакцинацииРезюме

Сосредоточение усилий на борьбе со столбняком новорожденных и матерей привело к тому, что меньше внимания стало уделяться заболеваемости столбняком и смертности от него среди мужчин. С 2007 года в 14 странах Африки к югу от Сахары расширилось участие населения в программах добровольного медицинского мужского обрезания, зарекомендовавшего себя как эффективное вмешательство, снижающее риск заражения вирусом иммунодефицита человека (ВИЧ) среди мужчин. В ходе анализа нежелательных явлений, вызванных этими программами, были определены 13 случаев заболевания столбняком, о которых Всемирной организации здравоохранения (ВОЗ) сообщили пять стран до марта 2016 года. Восемь больных скончались, и только для одного больного были получены сведения о пройденной вакцинации против столбняка. Среди более чем 11 миллионов человек, подвергнутых процедуре добровольного мужского обрезания, частота случаев заболевания столбняком была невелика. Тем не менее обнаружение этих случаев стало причиной анализа фактических данных об охвате вакцинацией против столбняка и уведомлениях о случаях заболевания в странах Африки к югу от Сахары. Дополнительно с этим анализом был проведен обзор литературных источников, относящихся к заболеванию столбняком (кроме неонатального) в Африке за период между 2003 и 2014 годом. Наибольшее количество случаев заболевания столбняком (кроме неонатального) на миллион жителей и наименьший зафиксированный охват вакцинацией столбнячным анатоксином пришлись на государства-члены Африканского региона ВОЗ. Охват третьей вакцинацией против дифтерии, столбняка и полиомиелита составил от 65 до 98% населения в 14 странах в 2013 году. Согласно результатам исследований, проводимых с участием пациентов больниц, диагноз столбняка (кроме неонатального) был поставлен 0,3–10,7% поступивших пациентов и среднее количество мужчин среди больных составило 71%. Выявление случаев заболевания столбняком после добровольного медицинского мужского обрезания свидетельствует о гендерном разрыве в заболеваемости столбняком, которая в непропорциональной мере затрагивает мужчин. Внедрение вакцинации против столбняка для мальчиков и мужчин в национальные программы должно являться приоритетом для достижения согласованности с задачей обеспечения всеобщего охвата услугами систем здравоохранения.

## Introduction

Tetanus is a rapidly progressing, painful disease with a high mortality rate, yet is inexpensive to prevent. Although tetanus toxoid was first licensed as a vaccine in 1937, tetanus remains a public health problem in many parts of the world and is often fatal, even within modern intensive care facilities.^1^^,^^2^ According to World Health Organization (WHO) recommendations, a series of three tetanus-toxoid-containing vaccine doses should be given in infancy, followed by booster doses at the age of school entry, in adolescence and in adulthood to induce longer-term immunity.^1^^,^^3^ WHO’s focus on the elimination of maternal and neonatal tetanus by 2015 led to vaccination strategies targeting women of reproductive age and infants.^4^^,^^5^ Less attention, however, has been given to the immunization of males after infancy. Data on child and adult vaccination coverage and tetanus incidence and mortality among men are limited.

Emerging reports of cases of tetanus following voluntary medical male circumcision in different sub-Saharan African countries drew our attention to the possibility of a gender disparity in tetanus morbidity that disproportionately affected men. In this paper we report a summary of the reported tetanus cases, together with a review of the evidence on tetanus vaccination coverage and case notification in sub-Saharan Africa, supplemented by a review of the literature on non-neonatal tetanus over the past 10 years.

## Emerging reports

### Context

Voluntary medical male circumcision is an effective intervention to reduce the risk of human immunodeficiency virus (HIV) acquisition among men. When the intervention is scaled-up, HIV incidence is reduced and costs are saved for health programmes and budgets.^6^ In 2007, WHO and the Joint United Nations Programme on HIV/AIDS recommended the intervention in countries with a high prevalence of HIV and historically low rates of male circumcision.^7^ By the end of 2015 over 11 million men had been circumcised through voluntary medical male circumcision programmes in 14 priority countries in eastern and southern Africa: Botswana, Ethiopia, Kenya, Lesotho, Malawi, Mozambique, Namibia, Rwanda, South Africa, Swaziland, the United Republic of Tanzania, Uganda, Zambia and Zimbabwe (unpublished data, WHO, 2016). As an elective procedure chosen by often healthy men to reduce future HIV risk, ensuring its safety is a priority. Three conventional surgical methods (dorsal slit, forceps-guided and sleeve resection) and two device methods (clamps or collars that remain in place for 1 week) have been used. WHO has recommended 10 standards for quality assurance, including infection prevention and control,^8^ and has encouraged each country to carry out adverse event surveillance, particularly when implementing new methods. WHO made an initial review of adverse events identified from voluntary medical male circumcision programmes in 2014 and continues to do so through post-market surveillance and country reports.

### Tetanus case reports

We examined summary reports of all tetanus cases reported to the national voluntary medical male circumcision programmes and submitted to WHO. Additional details were requested from ministries of health as needed. We identified reports of 13 cases of tetanus in which the client presented for care within 14 days of a voluntary medical male circumcision procedure; eight cases resulted in death (Table 1). The cases, recorded from April 2012 up to March 2016, were reported from five of the 14 priority African countries: Kenya, Rwanda, Uganda, the United Republic of Tanzania and Zambia.

**Table 1:** Key features of 13 cases of tetanus after voluntary medical male circumcision reported to the World Health Organization from 2012 to 2016

Procedure date	Country	Client’s age, years	Procedure method	Days to symptoms	Days to diagnosis	Days to death	Circumcision wound	Unclean substance applied to wound	Alternate exposure route on body
Mar 2016	Rwanda	34	Device	8	11	12	Clean	Unknown	No
Sep 2015	Rwanda	39	Device	Unknown	14	N/A	Clean	Unconfirmed	Yes
Mar 2015	Uganda	11	Surgery	7	10	12	Septic	Yes	No
Mar 2015	Uganda	19	Surgery	10	12	N/A	Clean	Unknown	Yes
Nov 2014	United Republic of Tanzania	18	Surgery	11	16	35	Septic	Yes	Unknown
Sep 2014	Uganda	32	Device	7	8	14	Septic	Unknown	Unknown
Sep 2014	Uganda	11	Surgery	11	12	17	Septic	Yes	Yes
Aug 2014	Kenya	15	Surgery	11	11	13	Septic	Yes	No
Aug 2014	Uganda	19	Device	11	12	14	Septic	Unknown	Unknown
May 2014	Rwanda	47	Device	12	12	N/A	Clean	Unconfirmed	Yes
Jun 2013	Uganda	18	Surgery	8	15	N/A	Clean	Unknown	Yes
Dec 2012	Zambia	12	Surgery	5	8	9	Septic	Yes	No
Apr 2012	Zambia	16	Surgery	12	12	N/A	Septic	Unknown	No

N/A: not applicable.

The circumcision methods included both conventional surgery (eight patients, of whom five died) and an elastic collar compression device method (five patients, of whom three died). The period from surgery or device placement to symptom onset ranged from 5 to 12 days, with a mean of 11.8 days to clinical diagnosis. Mean time to death was 15.8 days for the eight patients who died. Using a standardized case definition,^9^ 12 of the 13 cases were consistent with a causal association with male circumcision. Health-care providers who examined the patients for tetanus reported that the circumcision wound was septic in seven patients, whereas the same circumcision wound was noted to be clean in six patients at a circumcision follow-up visit before tetanus was diagnosed. It is possible that health-care providers unfamiliar with the appearance of circumcision wound healing may have misclassified the wound as septic. Alternatively, the infection could have occurred after the last circumcision visit or could have been from another injury. Five patients had other potential wound sites including injuries and infections of the lower limbs. A home remedy had been applied to the circumcision wound in five patients treated with surgery and possibly in two patients with devices. Hygiene conditions of the person or his home were noted to be poor in five patients. 

Nine of the 13 patients were adolescents (aged 10–19 years). All men who were working had outdoor-based occupations such as farming and brick-making.

Based on records or patients’ recall, only one of the 13 patients had a history of tetanus vaccination. However, three patients had received tetanus toxoid immediately before the procedure; one patient because pre-surgical vaccination was the routine practice of the clinic that provided the circumcisions and two patients because the programme instructions were updated in 2015. One of these patients died after device-type circumcision.

## Non-neonatal tetanus risk

### Tetanus notifications

These emerging reports of tetanus cases after voluntary male circumcision prompted us to review the global data on non-neonatal tetanus. We examined the official WHO database for country-specific annual numbers of reported tetanus cases.^10^ Although non-neonatal tetanus (i.e. cases in patients over the age of 28 days) is not a reportable condition, some countries report both neonatal and non-neonatal cases. Neonatal tetanus reporting to the WHO notifiable surveillance system has very low notification efficiency, ranging from 3% to 11%,^11^ and cases of non-neonatal tetanus have not been routinely reported by most countries. Due to this differential reporting, comparisons across individual countries and WHO regions were difficult. As an indication, however, in 2013 the WHO African Region had the highest reported number of non-neonatal tetanus cases at 4.0 per million population (3732 cases among the total regional population of 927 370 712; Table 2), followed by the South-East Asia Region at 1.9 per million population (3432 cases among 1 855 067 643 people). Of the 12 African countries reporting any cases of non-neonatal tetanus, Uganda – the only country among them implementing voluntary medical male circumcision for HIV prevention – had the highest number of non-neonatal tetanus cases at 67.1 per million population (2522 cases among 37 578 880 people; Table 3). 

**Table 2:** Cases of non-neonatal tetanus reported in 2013, by region of the World Health Organization

Region	Population^a^	No. of reported tetanus cases			No. of non-neonatal cases per 1 000 000 population^c^
		All	Neonatal	Non-neonatal^b^	
African Region	927 370 712	6 508	2 776	3 732	4.0
Region of the Americas	966 494 922	457	20	437	0.5
Eastern Mediterranean Region	612 580 145	1 513	1 280	233	0.4
European Region	906 995 743	102	0	102	0.1
South-East Asia Region	1 855 067 643	4 153	721	3 432	1.9
Western Pacific Region	1 857 588 557	2 127	679	1 448	0.8

^a^ 2013 World Health Organization (WHO) mid-year country population estimate.

^b^ Non-neonatal tetanus (occurring after the first 28 days of life) is not a reportable condition and therefore many countries do not report this figure to WHO.

^c^ Due to reporting differences between countries, this number should not be interpreted as the incidence. It is provided as an indication of the scale of the problem; direct comparisons between Regions should not be made.

Source: World Health Organization, online database.^11^

**Table 3:** African countries reporting any cases of non-neonatal tetanus, 2013

Country	Population^a^	No. of reported tetanus cases			No. of non-neonatal cases per 1 000 000 population^c^
		All	Neonatal	Non-neonatal^b^	
Angola	21 471 617	360	33	327	15.2
Burkina Faso	16 934 838	27	0	27	1.6
Democratic Republic of the Congo	67 513 680	1 359	1 327	32	0.5
Liberia	4 294 078	8	0	8	1.9
Madagascar	22 924 850	556	8	548	23.9
Mali	15 301 650	37	12	25	1.6
Mauritania	3 889 882	4	0	4	1.03
Niger	17 831 269	71	1	70	3.9
Nigeria	173 615 344	556	468	88	0.5
Senegal	14 133 280	78	4	74	5.2
South Sudan	11 296 174	32	25	7	0.6
Uganda^d^	37 578 880	2 928	406	2 522	67.1

^a^ 2013 World Health Organization (WHO) mid-year country population estimate.

^b^ Non-neonatal tetanus (occurring after the first 28 days of life) is not a reportable condition and therefore many countries do not report this figure to WHO.

^c^ Due to reporting differences between countries, and likely data quality issues, this number should not be interpreted as the incidence. It is provided as an indication of the scale of the problem; direct comparisons between countries should not be made.

^d^ Implementing voluntary medical male circumcision for human immunodeficiency virus prevention.

Source: World Health Organization, online database.^11^

### Tetanus vaccination coverage

**Fig. 1:**
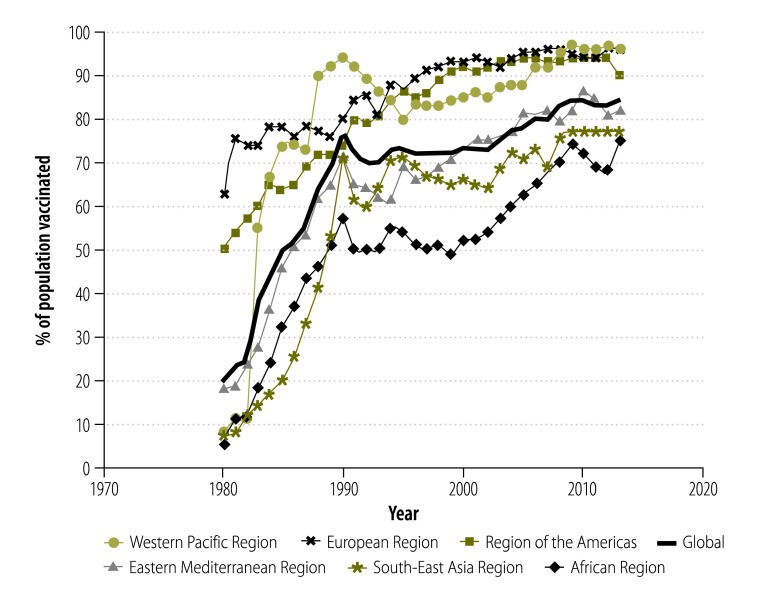
**Coverage of third dose of diphtheria–pertussis–tetanus (DTP3) vaccine from 1980 to 2013, by region of the World Health Organization **

Source: World Health Organization, online database.^13^

We also analysed the global joint WHO and United Nations Children’s Fund database^12^ for official data on countries’ coverage of the third dose of infant diphtheria–pertussis–tetanus (DPT3) vaccine from 1980 to 2013, grouped by WHO region. Coverage of fourth, fifth and sixth booster doses are not routinely reported. In 1980, when WHO started collecting data on DTP3 vaccination coverage, all regions apart from the Americas and European had coverage under 20%. Since then, global coverage of DTP3 vaccination increased steeply (Fig. 1) and by 2013 the lowest regional coverage was 75% in the WHO African Region and the global average was 86%.

**Fig. 2:**
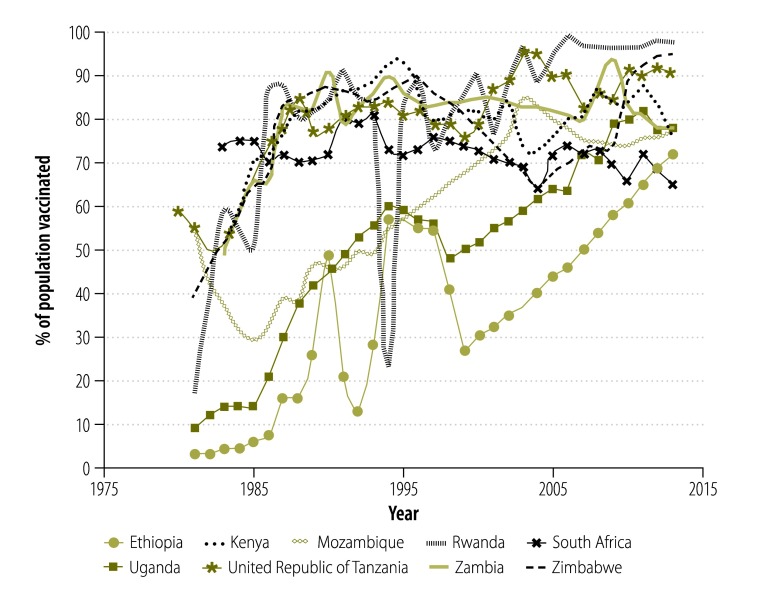
**Coverage of third dose of diphtheria–pertussis–tetanus (DTP3) vaccine in nine sub-Saharan African countries implementing voluntary medical male circumcision, from 1980 to 2013**

Note: Countries included have reported a case of tetanus after the procedure or had low DTP3 coverage (≤ 75% in at least 2 years since the year 2000).

Source: World Health Organization, online database.^13^

Fig. 2 shows DTP3 vaccination coverage in the nine African countries implementing voluntary medical male circumcision that have reported a case of tetanus after the procedure or that have low DTP3 coverage (≤ 75% coverage in at least 2 years since the year 2000). Among these countries, the DTP3 vaccination coverage reached 80% on average in 2005 and ranged from 65% in South Africa to 98% in Rwanda in 2013. As far as we are aware, most of the 14 priority countries for voluntary medical male circumcision have no policy for vaccinating males against tetanus after infancy.

### Literature review

To supplement evidence from the surveillance data, we conducted a literature review to gather additional information on non-neonatal tetanus. We searched the PubMed database using the MeSH terms “tetanus” and “Africa South of the Sahara”. We restricted the results to human studies in the period 2003–2014 and included all studies on adolescents and adults in any language. We excluded studies related to neonatal tetanus as well as case reports. At a minimum we reviewed all abstracts, including English versions of non-English publications, and obtained the full text of selected manuscripts.

Our database search resulted in 259 studies, of which 28 were on non-neonatal tetanus; we included a further four studies identified from references or by colleagues. These 32 studies^13^^–^^44^ originated from 10 African countries; all were based on hospital inpatient cases. Their key features are summarized in Table 4. Across the studies, a median of 71% of patients admitted to hospital with tetanus were men. The median age of tetanus patients (estimated from the mean and median ages, as reported in the articles) was 32.7 years. Non-neonatal tetanus cases comprised 0.3–10.7% of all hospital admissions, and in one Côte d’Ivoire study, surgery-related tetanus constituted 11.0% of all 273 non-neonatal tetanus admissions. The median case fatality rate from non-neonatal tetanus was 44.0% and ranged from 0% of 12 inpatients in a small Nigerian study to 80.0% of 175 children in another Nigerian study. Ten studies listed lower limb injuries as one of the main causes of tetanus, and two studies mentioned male circumcision among their infection sources. Based on the eight studies reporting vaccination status, high proportions of tetanus inpatients had not been vaccinated (range: 83−100%) or had unknown vaccination status. 

**Table 4:** Summary of hospital studies of non-neonatal tetanus in sub-Saharan Africa countries, 2003 to 2014

Reference	Country	Study period	Population	Total no. of hospital admissions	Non-neonatal tetanus cases			
					No.	Average age,^a,b^ years	Male, %	Case fatality rate, %
Sawe et al. (2014)^14^	United Republic of Tanzania	2009–2011	ICU admissions at four tertiary hospitals	5 627	135	–	–	71.0
Muteya et al. (2013)^15^	Democratic Republic of the Congo	2005–2009	All tetanus admissions	1 029	22	39.4^a^	95.2	52.4
Traoré et al. (2013)^16^	Guinea	2001–2012	Tetanus cases at all hospitals in Conakry	8 649	239	–	73.0	75
Oshinaike et al. (2012)^17^	Nigeria	2006–2011	Tetanus admissions, age > 10 years	9374	218	29.4^a^	75.6	56.2
Bankole et al. (2012)^18^	Nigeria	2000–2009	Adult tetanus admissions	78 009	190	30.4^a^	75.0	16.3
Amare et al. (2012)^19^	Ethiopia	2001–2009	Tetanus admissions, age ≥ 13 years	–	68	33.8^a^	77.9	35.3
Minta et al. (2012)^20^	Mali	2004–2009	Tetanus admissions, age ≥ 15 years	1 839	119	32.9^a^	84	46.2
Aba et al. (2012)^21^	Côte d'Ivoire	2003–2008	Surgical tetanus cases	273^c^	29	36.0^a^	79	45.0
Amare et al. (2011)^22^	Ethiopia	1996–2009	Tetanus admissions, age ≥ 13 years	–	171	33.0^a^	75.4	38.0
Ugwu and Ugwu (2011)^23^	Nigeria	1999–2008	Children after intramuscular injection	175^c^	12	–	60.0	80.0
Akhuwa et al. (2010)^24^	Nigeria	2005–2008	Post-neonatal tetanus cases	–	18	5.8^a^	77.0	5.9
Fawibe (2010)^25^	Nigeria	2002–2006	Adult tetanus admissions	3 514	41	33.0^a^	85.7	57.1
Tadesse et al. (2009)^26^	Ethiopia	2003–2008	Adult tetanus admissions	–	29	35.0^a^	65.5	41.4
Dao et al. (2009)^27^	Mali	2001–2004	All tetanus admissions	9 65	57	39.0^a^	69.0	38.9
Zziwa et al. (2009)^28^	Uganda	2005–2008	All tetanus admissions	25 118	145	–	66.0	38.4
Chukwubike et al. (2009)^29^	Nigeria	1996–2005	Tetanus admissions, age ≥ 16 years	8 762	86	30.2^a^	58.1	42.9
Ajose and Odusanya (2009)^30^	Nigeria	2004–2006	Adult tetanus admissions	–	164	29.6^a^	75.6	70.1
Towey and Ojara (2008)^31^	Uganda	2005–2006	All ICU admissions	218	17	–	–	47.0
Soumaré et al. (2008)^32^	Senegal	1999–2006	Post-circumcision tetanus at infectious diseases clinic	27 295	1 291	9.0^a^	n/a	7.4
Onwuekwe et al. (2008)^33^	Nigeria	1999–2003	All tetanus admissions	–	12	29.8^a^	58.0	0.0
Komolafe et al. (2007)^34^	Nigeria	1995–2004	Adult tetanus admissions	–	79	–	70. 9	45.0
Sanya et al. (2007)^35^	Nigeria	1990–2001	Adult tetanus admissions	–	288	36.1^a^	69.3	63.9
Melaku et al. (2006)^36^	Ethiopia	1985–2000	All tetanus admissions	3 548	146	32.3^a^	69.9	49.3
Ndour et al. (2005)^37^	Senegal	1999–2002	Tetanus after intramuscular injection	–	46	34.5^a^	63	60.8
Amsalu et al. (2005)^38^	Ethiopia	1989–1998	Children with tetanus diagnosis	–	51	9.0^b^	54	31.4
Soumaré et al. (2005)^39^	Senegal	Mar–Sep 2002	Children with tetanus, age 1–15 years	757	40	8.8^a^	75.0	8.0
Soumaré et al. (2005)^40^	Senegal	Sep–Dec 2002	Tetanus admissions, age > 4 years	–	30	36.0^a^	70.0	26.7
Ojini and Danesi (2005)^41^	Nigeria	1990–1999	Tetanus admissions, age ≥ 10 years	–	349	29.8^a^	66.0	37.0
Seydi et al. (2005)^42^	Senegal	2001–2003	Tetanus admissions, age > 28 days	4 123	440	20.0^a^	70.7	22.0
Mchembe and Mwafongo (2005)^43^	United Republic of Tanzania	Jan–Dec 2004	Tetanus admissions	–	22	–	91.0	72.7
Tanon et al. (2004)^44^	Côte d'Ivoire	1985–1998	All tetanus admissions	62 313	1 870	28.0^b^	71.0	31.9
Hesse et al. (2003)^45^	Ghana	1994–2001	All tetanus admissions	–	158	32.7^a^	76.6	50.0

ICU: intensive care unit.

^a^ Mean age.

^b^ Median age.

^c^ Hospitalized tetanus cases.

Note: Dashes indicate data not available or not applicable.

## Discussion

Our investigation into tetanus cases identified through voluntary medical male circumcision programmes and an analysis of available global data highlights a gender gap in tetanus morbidity that disproportionately affects men. The occurrence of tetanus following voluntary medical male circumcision was rare – with 13 cases reported from programmes that have conducted over 11 million procedures by the end of 2015 – and may be no higher than the background incidence of tetanus among men in these countries.

National tetanus case reporting and hospital studies suggest that the incidence of non-neonatal tetanus may be substantial in some countries in the WHO African Region, and that the majority of inpatient cases are among men. As non-neonatal tetanus is not reportable in most low- and middle-income countries, the underlying tetanus burden may be higher than we found. The efforts worldwide towards the goal of elimination of maternal and neonatal tetanus has reduced tetanus incidence and mortality in those groups through vaccination during pregnancy and clean delivery and cord-care practices.^5^ However, adolescent and adult men seem to have been largely missed by vaccination programmes, as implementation of the WHO-recommended fourth to sixth doses of tetanus vaccine to adolescents and adults has been limited. Only one of the 13 tetanus cases reported by voluntary medical male circumcision programmes had a known history of tetanus vaccination. Three clients received a dose of tetanus-toxoid-containing vaccine immediately before male circumcision; two recovered from the tetanus infection and one died.

We found that infant tetanus-toxoid-containing vaccine coverage levels in the African Region as a whole, and in some countries in particular, were historically low, although they have increased greatly since 1980. Countries with a history of low coverage of infant immunization, and no national policy or practice for tetanus vaccine administration to adolescent or adult men, could be expected to have a large proportion of adolescent and adult men who are insufficiently protected against tetanus infection. These men are therefore at risk of acquiring tetanus from injuries or surgical procedures. Voluntary medical male circumcision programmes must maintain quality assurance standards, including infection control, and inform clients of the risk of tetanus if the circumcision wound is exposed to substances that might be contaminated with *Clostridium tetani* spores, including home remedies.^9^

Incorporating tetanus vaccination into voluntary medical male circumcision programmes should be seen as a priority. In vaccine-naïve individuals, two tetanus-toxoid-containing vaccine doses spaced 4 weeks apart are needed, with a further 2-week interval before performing the procedure. Providing a booster dose at least seven and ideally 14 days before voluntary medical male circumcision in individuals who are not fully vaccinated may induce partial immunity; an additional dose given after the procedure would also provide longer-term immunity. In the long term, tetanus vaccination, which costs less than 1 United States dollar, should be included in school-based programmes for both girls and boys at ages 4–7 years and 12–15 years, with additional targeting of adults to ensure long-lasting protection from this disease.

Some of the limitations of our analyses are that first, many countries do not report non-neonatal tetanus cases to WHO. This reporting difference may lead to the burden of tetanus appearing greater in some countries or regions than in others. For this reason, we have limited our interpretation of these data to an indication of broad trends in tetanus rates and not an analysis of incidence. Second, our review of the literature was limited to one database. However, we believe it was sufficient to gain a general picture of the burden of non-neonatal tetanus in sub-Saharan Africa.

In conclusion, although both men and women are at risk of tetanus infection, our analyses show that there is an underlying burden of tetanus among adolescent and adult men who have been largely missed by vaccination programmes. Incorporating tetanus-toxoid-containing vaccine for boys and men into national immunization programmes should be encouraged to reduce the morbidity and mortality from this preventable disease. Enhanced personal hygiene and wound-care practices should also be emphasized after voluntary medical male circumcision. Elevating non-neonatal tetanus to a reportable condition would fill the knowledge gap about the incidence. The convergence of cost–effective solutions to two public health problems affecting men – HIV and tetanus – offers an opportunity for service synergies and enhanced health equity. Addressing this gender gap, and aligning with goals for universal health coverage and access to vaccines for all, should be an explicit policy goal for national health programmes and relevant partners.
